# Fluoride Exposure and Salivary Glands: How Is Glandular Morphology Susceptible to Long-Term Exposure? A Preclinical Study

**DOI:** 10.3390/jcm11185373

**Published:** 2022-09-13

**Authors:** José Mário Matos-Sousa, Leonardo Oliveira Bittencourt, Maria Karolina Martins Ferreira, Vinicius Ruan Neves dos Santos, Karolyny Martins Balbinot, Sérgio Alves-Júnior, João de Jesus Viana Pinheiro, Senda Charone, Juliano Pelim Pessan, Rafael Rodrigues Lima

**Affiliations:** 1 Laboratory of Functional and Structural Biology, Institute of Biological Sciences, Federal University of Pará, Belém 66075-110, PA, Brazil; 2 School of Dentistry, Federal University of Pará, Belém 66075-110, PA, Brazil; 3 Department of Preventive and Restorative Dentistry, School of Dentistry, São Paulo State University, Araçatuba 14801-385, SP, Brazil

**Keywords:** fluoride, sodium fluoride, salivary glands

## Abstract

Despite a strong body of evidence attesting to the effectiveness of fluoride (F) in preventing and controlling caries, some studies have sought to investigate the influence of F exposure on the salivary glands, organs that are essential for the maintenance of cavity homeostasis through salivary production, finding that exposure to F can cause biochemical and proteomic changes. Thus, this study aimed to investigate the morphological effects of prolonged exposure to F on the salivary glands of mice, at concentrations that would correspond to optimally fluoridated water (suitable for human consumption) and to fluorosis-endemic regions. Twenty-four male mice (*Mus musculus*) were divided into three groups, according to F levels in the drinking water: 0 (control), 10, or 50 mg F/L, with an exposure period of 60 days. The glands were morphometrically analyzed for the total acinar area, parenchyma area, and stromal area, as well as for the immunohistochemical analysis of myoepithelial cells. The results showed that prolonged exposure to F at 10 mg F/L did not promote significant changes in the morphometry of the salivary glands of mice, which reinforces the safety of the chronic use of F in low doses.

## 1. Introduction

Fluorine is a non-metallic element with high electronegativity, which can be found naturally in the forms of fluoride (F). The exposure to this ion can occur both naturally (through volcanic activity, coal combustion, dissolution of minerals, and marine aerosols) and anthropogenically (when F is released as waste from industrial activity or from manufactured products) [1,2]. Due to its unquestionable effects on caries prevention and control, F is also incorporated into vehicles available at a population level (water, salt, milk, sugar, and supplements), as well as at an individual level for professional (gels, foams, and varnishes) and home application (dentifrices and mouthwashes), which are known to contribute to the overall systemic exposure to fluoridated compounds [3].

Considering the possibility of bioaccumulation in organic systems, previous studies indicate that F ingested at excessive doses is capable of causing damage to the central nervous system [4], reduced immunity [5], male reproductive system dysfunction [6], liver damage [7], alterations in the antioxidant systems of kidney [8], and skeletal and dental manifestations [9,10]. Furthermore, F may interact with various cellular processes, such as gene expression, cell cycle, proliferation and migration, respiration, metabolism, ion transport, vesicular transport, oxidative stress, and cell death [11].

Moreover, F has been shown to impair the metabolism of key organs that ensure the physiological balance of the oral environment, including the salivary glands. These are secretory organs that contribute to the homeostasis of the oral cavity, and whose products play a substantial role in the regulation of physiological properties and maintenance of soft tissue integrity [12,13]. Total salivary production and composition vary according to different physiological conditions [14]. The major salivary glands, represented by three pairs of glands (parotid, submandibular, and sublingual), produce on average 90% of the saliva secreted in the oral cavity, while the remainder is produced by minor salivary glands dispersed in the mucosa [15]. Thus, total saliva becomes a complex of multiglandular secretions composed of gingival fluid, desquamated epithelial cells, microorganisms, products of bacterial metabolism, food remnants, leukocytes, and mucus from the nasal cavity and the pharynx [16].

In a previous study, the biochemical parameters of the submandibular salivary glands of mice under F exposure were evaluated through proteomic analysis, showing changes in the protein profile of the groups exposed to F at 10 mg F/L or 50 mg F/L, which were especially related to the structural constituents of the cytoskeleton and actin-myosin filaments [17]. Furthermore, a similar study on the parotid salivary glands reached similar results in the proteomic analysis, but also reported alterations in proteins associated with the anatomical structure of the glands [17,18]. Based on the aforementioned studies, the question on the effects of these biochemical alterations on the morphological structure of the salivary glands can be raised. Thus, considering that possible tissue-level repercussions of biochemical, proteomic, and genotoxic changes in salivary glands caused by prolonged exposure to F have not yet been addressed, there is a need to investigate the effects of F on the morphology of the salivary glands, thus seeking a better understanding of the possible risks to human health.

Within the context above, this study aimed to investigate the effects of systemic and chronic exposure to F on the morphometric parameters of the major salivary glands of mice. The study’s hypothesis was that the molecular damages caused by F exposure reported in the literature would be associated with histological alterations in the salivary glands of the exposed animals.

## 2. Materials and Methods

### 2.1. Experimental Animals

The experimental procedures were performed after approval by the Ethics Committee on Experimental Animals of the Federal University of Pará (UFPA), project No. 7360181219, following the recommendations of the NIH Guide for Care and Use of Laboratory Animals. A total of 24 male Swiss albino mice (21 days old) were randomly divided into three groups of 8 animals each, which were fed ad libitum with chow and water, and housed inside an air-conditioned room, with a 12 h light/dark cycle. For 60 days, all animals received deionized water containing 10 mg F/L and 50 mgF/L (as NaF, Sigma-Aldrich—San Luis, AZ, USA), to mimic chronic F ingestion from water by humans at concentrations corresponding to ≅2 mg F/L (roughly equivalent to the concentration of drinking water)) and 10 mg F/L (equivalent to water from areas endemic to fluorosis). This concentration is justified by the metabolism of rodents being 5 times higher than that of humans [19,20,21]. The control group received only deionized water without NaF for the same period. The weight of the animals and the volume of water ingested per cage were measured weekly. Other studies by our research group used this same exposure protocol [17,18,19], which found increased fluoride concentrations in the submandibular and parotid glands, and in blood plasma. The results from these initial studies were that F levels in the group receiving the highest exposure dose (50 mg F/L) were significantly higher (0.19 ± 0.01 µg/mL) than the levels in the control group (0.05 ± 0. 01 µg/mL) in the submandibular glands [17], as well as in the parotid glands, with similar results as the levels in the group receiving the highest exposure dose (50 mg F/L) were significantly higher (0.14 ± 0.01 µg/mL) than the levels in the control group (0.06 ± 0.01 µg/mL) [18]. Furthermore, an increase in plasma fluoride concentration, finding statistically higher results in the groups exposed to 10 mg/L NaF (0.122 µg F/mL) and 50 mg/L NaF (0.142 µg F/mL) when compared to the control group (0.081 µg F/mL) mL) [19]. This led us to devise the present experimental design.

### 2.2. Euthanasia and Salivary Glands Collection

**Figure 1:**
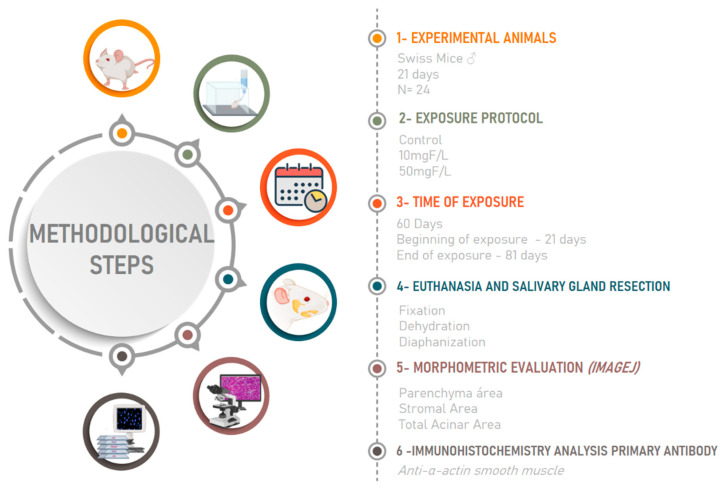
Experimental protocol of the study. In 1, description of experimental animals; in 2, the method of exposure to fluoride (F) at 0, 10 or 50 mg F/L; in 3, time of exposure and age at which experiment started and ended; in 4, euthanasia, salivary glands resection and histological processing steps; in 5, morphometric evaluation; and 6, immunohistochemical analyses.

All animals were anesthetized with a solution of ketamine hydrochloride (90 mg/kg) and xylazine (9 mg/kg) for the perfusion protocol [22]. Then, the collections of the glands were performed for histological analysis, as described below and represented in Figure 1.

### 2.3. Histopathological and Morphological Evaluations

After the surgical process for the collection of the parotid, submandibular and sublingual salivary glands, all samples were immersed in 10% formalin for 48 h for further tissue analysis.

To assess and quantify morphological and tissue changes, morphometric and immunohistochemical analyses were performed. For this, after the period of fixation of the samples in formalin, the glands of each animal were post-fixed in 6% formaldehyde until processing. The glands were dehydrated in increasing solutions of ethanol (70%, 80%, 90%, absolute 1, and absolute 2) cleared in xylene, and included in Paraplast for subsequent 5 µm-thick sections.

Twenty sagittal sections of the glands previously included in Paraplast were made, with a thickness of 5 μm, and then stained with Hematoxylin and Eosin. To perform this analysis, images were taken by a color digital camera (Cyber-Shot DSC W-230, Sony, Tokyo, Japan) coupled to a microscope (Eclipse E200, Nikon, Tokyo, Japan; at a magnification of 40×) of 4 random sagittal sections of the glands being evaluated, on average, 4 fields from each section. The tissue morphometric evaluation variables, expressed in µm^2^, were: Total Acinar Area (TAA), Total Duct Area (TDA), Parenchyma Area (PA), and Stromal Area (SA) [23,24].

Variable values were obtained using a digital image analyzer (ImageJ software, v. 1.53, NIMH, NIH, Bethesda, MD, USA, http://rsbweb.nih.gov/ij/ (accessed on 1 June 2022).

#### Immunohistochemistry Analysis

The immunohistochemical analysis was performed because myoepithelial cells (present around the acini of the salivary glands) are difficult to observe when stained by Hematoxylin and Eosin. Due to the great importance of this group of cells for the functioning of the salivary glands, immunohistochemistry was used to overcome the limitations of conventional staining techniques. For this analysis, the slides were deparaffinized in xylene and hydrated in decreasing concentrations of alcohol. Antigen retrieval was performed in citrate buffer (pH 6.0) in a Pascal pressure chamber (Dako, Carpinteria, CA, USA) for 30 s. Then, they were immersed in 6% H_2_O_2_ and methanol at a ratio of 1:1 for 30 min to inhibit the activity of endogenous peroxidase. Non-specific binding was blocked with 1% bovine serum albumin (BSA) (Sigma-Aldrich Corp., St. Louis, MI, USA) in phosphate-buffered saline (1× PBS) for 1 h.

After this step, primary anti-α-smooth muscle actin antibodies (DAKO, 1:350) to actin filaments of myoepithelial cells were incubated for 1 h. Then, the slides were incubated for 30 min with the Immunohistoprobe plus detection system (Advance Biosystems, San Francisco, CA, USA). Diaminobenzidine (DAB) (ScyTek, Logan, UT, USA) was used as a chromogen. Slides were counterstained with hematoxylin (Sigma^®^), dehydrated, divinized, and mounted with Permount (Fisher Scientific, Fair Lawn, NJ, USA).

The immunostaining was carried out by evaluating the area measurement (μm) and a fraction (%) of the marking of the analyses performed. Brightfield images of 3 randomly selected areas from each sample were acquired using the same magnifications (40×).

The areas revealed by diaminobenzidine (DAB) were separated and segmented using the “color deconvolution” tool (Gabriel Landini, http://www.dentistry.bham.ac.uk/landinig/software/software.html (accessed on 1 June 2022) available in the ImageJ software (NIMH, NIH, Bethesda, MD, USA). After image segmentation, the total area and total staining fraction were measured. The immunostaining differences found in the studied groups were analyzed.

### 2.4. Statistical Analyses

Data were statistically analyzed using the GraphPad Prism v. 8.0 software (San Diego, CA, USA), using one-way ANOVA (for parametric data) or Kruskal–Wallis test (for non-parametric data), and Tukey’s post hoc test, assuming a statistical significance value of *p* < 0.05. Parametric results are expressed mean ± standard error (Supplementary Table S1), while non-parametric results were expressed as median, interquartile range (Supplementary Table S2).

## 3. Results

### 3.1. Long-Term F Exposure Is Not Able to Alter the Morphology of Mice Salivary Glands 

**Figure 2:**
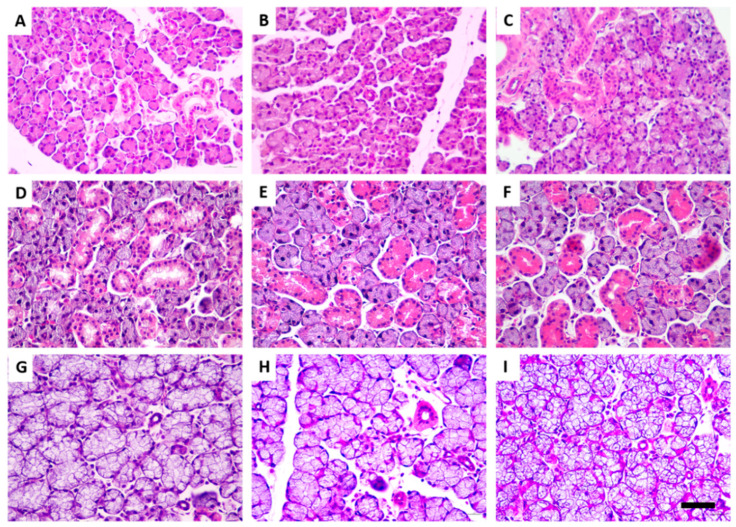
Effects of 60-day exposure to 0, 10, or 50 mg F/L from the drinking water on the histologic characteristics of the salivary glands of mice. Photomicrographs were obtained from the parotid (**A**–**C**), submandibular (**D**–**F**), and sublingual (**G**–**I**) glands, which were stained with hematoxylin and eosin. From left to right, exposure to 0 mg F/L (control), 10 mg F/L, and 50 mg F/L. 50 µm scale bar. (*n* = 8 animals/group).

The three major salivary glands showed a pattern of morphological normality, with intact acini and grouped in lobes, close to the ducts, with uniformity of the areas of parenchyma and stroma for the three experimental groups, as shown in Figure 2.

**Figure 3:**
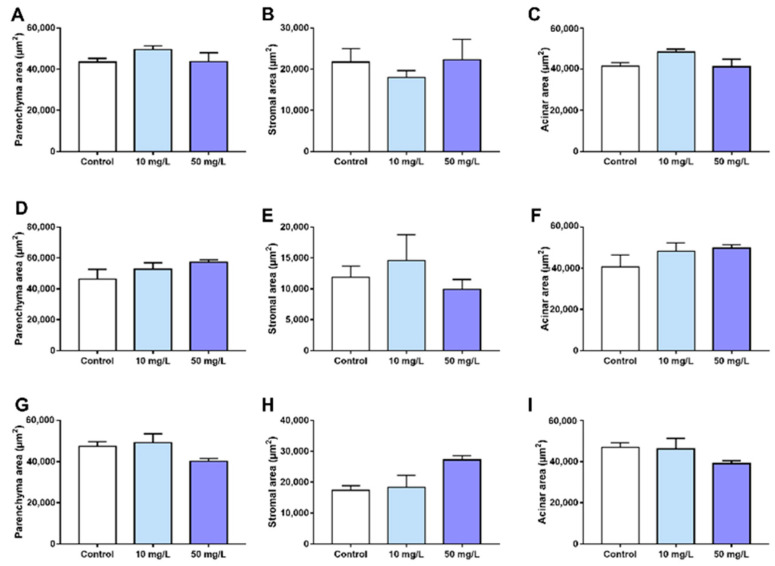
Effects of 60-day exposure to 0, 10, or 50 mg F/L from the drinking water on the morphometric parameters of the mice salivary glands. Graphs represent the morphometric analyses of the parotid glands (parenchyma, stromal, and acini areas represented by (**A**–**C**), respectively), submandibular glands (parenchyma, stromal, and acini areas represented by (**D**–**F**), respectively), and sublingual glands (parenchyma, stromal, and acini areas represented by (**G**–**I**), respectively). The results are expressed as mean ± standard error of mean. Different superscript letters indicate statistical differences among the groups. The absence of letters indicates that there was no statistical difference. One-way ANOVA and Tukey’s post hoc test. (*p* < 0.05, *n* = 8 animals/group).

For the quantitative analyses (Figure 3), no statistically significant differences were observed among the three groups regarding the measured areas of parenchyma (Figure 3A), stroma (Figure 3B), and total acinar area (Figure 3C) of the parotid glands.

A similar trend was observed for the submandibular glands, i.e., no statistical differences among the groups, considering the parenchyma area (Figure 3D), stromal area (Figure 3E), and total acinar area (Figure 3F).

For the sublingual glands, on the other hand, despite no statistical differences were observed among the three groups for the parenchyma area (Figure 3G) and the acinar area (Figure 3I), exposure to 50 mg F/L water led to significant increases in the stromal area (Figure 3H) compared with the groups exposed to 0 (control) or 10 mg F/L drinking water.

### 3.2. Long-Term F Exposure Did Not Affect the Immunostained Area Fraction of the Smooth Muscle Actin Filaments in Myoepithelial Cells of Mice Salivary Glands

**Figure 4:**
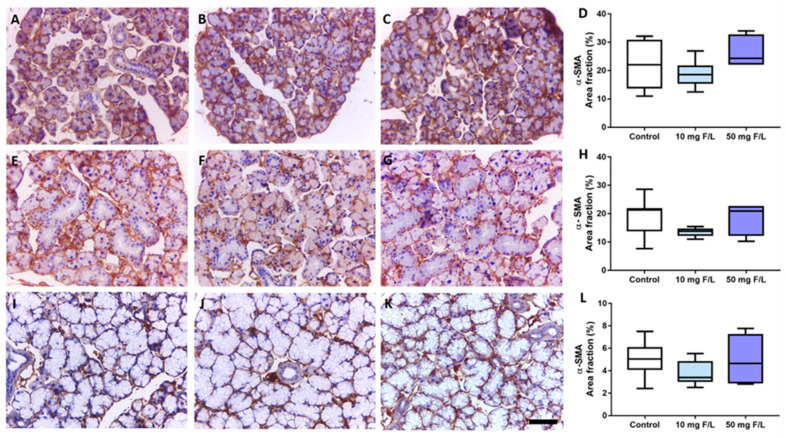
Effects of 60 days exposure to 0, 10, or 50 mg F/L on smooth muscle actin immunostaining area fraction in mice salivary glands, represented by photomicrographs of each group and graphs of the area fraction analysis. Letters (**A**–**D**) represent the parotid gland; (**E**–**H**), the submandibular gland; (**I**–**L**), the sublingual gland. From left to right (columns), control, 10 mg F/L, and 50 mg F/L groups, and area fraction graphs. Brown areas indicate positive labeling for the antibody by DAB reaction, while blue areas are counterstained by Harris’ hematoxylin. Results are expressed as median and interquartile range. Different superscript letters indicate a statistical difference between the groups. The absence of letters indicates that there was no statistical difference. Kruskal–Wallis test, followed by Tukey’s post hoc test, *p* < 0.05. 50 µm scale bar. (*n* = 8 animals/group).

The immunostaining pattern was fairly uniform among the control, 10 mg F/L and 50 mg F/L groups, showing a morphological normal pattern of myoepithelial cells, as presented in Figure 4. No statistical differences were observed among the groups (0, 10, or 50 50 mg F/L) regarding the parotid (Figure 4D), the submandibular (Figure 4H), and the sublingual (Figure 4L) glands.

## 4. Discussion

This study gathered morphological evidence about long-term exposure to F, and the repercussions on the salivary glands of adult mice. Our findings pointed out that F, under the present experimental conditions, does not pose a significant threat to the major salivary glands’ morphological organization in adult organisms, especially considering the data from the glandular functional unit, the acini, and the myoepithelial cells.

The F concentrations adopted were based on previous studies investigating F exposure from the drinking water, which somehow mimicked the F concentrations to which humans may be exposed to [21,25], then, from the known mean human ingestion doses of F, the resulting concentrations for exposure in this study were 10 mg F/L and 50 mg F/L [20].

In previous studies, we showed that systemic and prolonged exposure to F at 10 mg F/L and 50 mg F/L resulted in significant increases in F bioavailability in the blood plasma at both concentrations, however, only higher concentrations increased the levels of F in the parotid and submandibular glands of mice [17,18,19]. Despite these differences in F distribution among different organs, this evidence ensures the reliability in establishing a dose–response in our experimental protocol. Furthermore, it is worth mentioning that the exposure concentrations used were still above the optimal indication values [26].

In our morphometric results, we found an increase in the stromal area in the sublingual glands of animals exposed to 50 mg F/L, while no statistical differences were observed among the groups for this parameter for the other glands. In this context, it is important to emphasize that although the major salivary glands are fundamentally involved in the same role (i.e., saliva production), they have different morphological development and organization. The parotid gland has its terminal acini-like secretory units consisting of serous cells. The submandibular gland, on the other hand, is characterized as mixed, with serous predominance, but with the presence of mucous terminals. The sublingual glands have their composition substantially formed by tubular mucosal terminals, with the presence of some serous semilunas [27]. These structural differences may help to explain the increase in the stromal area of the sublingual gland of mice exposed to 50 mg F/L compared to the other glands.

Considering the peculiarities of the morphology of the salivary glands, the myoepithelial cells (which are attached to the basal lamina of the serous acinous secretory units) structurally resemble smooth muscle cells, and are composed of filament bodies of actin, myosin, and dense bodies, containing a central body with its nucleus and four to eight processes surrounding the secretory unit, as well as the proximal portion of the duct system. These cells are contractile, and their function is to facilitate and better promote the excretion of the secretory product from the acini glands to the duct system [27,28]. This ability is mediated by alpha-actin filaments, which are used as good cell markers for this cell population. Our findings showed that chronic exposure to F is not associated with the modulation in the immunostaining of smooth muscle actin filaments in the salivary glands, suggesting, therefore, that no impairment in salivary flow might be caused by F at the levels tested.

The functional and structural maintenance of the glands, demonstrated in our morphometric results, is essential to oral cavity homeostasis, considering that saliva contributes to the maintenance of a wide range of physiological needs. In the digestive tract, for example, saliva plays an important role in the digestive process and in the protection of gastric cells. In the oral cavity, saliva participates in mastication, speech, swallowing, and tissue lubrication, in addition to having antibacterial, antifungal, and antiviral activity. It also has buffering capacity and acts as a carrier of essential ions, such as calcium, phosphate, and fluorine itself, which have an essential role in enamel remineralization [29,30].

In this perspective, a recent study from our group showed that F exposure at the same concentrations as in the present work, during the pre- and post-natal periods, elicited morphological changes in the duct area of the parotid and submandibular glands in animals exposed to 50 mg F/L [31]. These findings prompted the present investigation, which assessed the morphological changes of the salivary glands of exposed mice from the end of the lactation period (21st day after birth), until the adult phase (at 81 days of life) [32]. Therefore, the exposure period of 60 days occurred during the stage of differentiation of the glands until the consolidated adult phase [28]. Differently from the prenatal studies [31], in this model of postnatal exposure to F no morphological changes were found. This different response suggests that susceptibility to damage is age-dependent and that the salivary glands show greater resistance to morphological damage that could be triggered by F after their full development.

The trend above, however, should be viewed with caution. Scientific evidence suggests that excess F leads to increases in reactive oxygen species production, accelerated consumption of antioxidant enzymes, and accumulation of lipid peroxidation products [33,34]. Previous studies have also shown that F is able to modulate oxidative biochemistry, such as increased GSH and TBARS, in addition to genotoxic changes in the submandibular gland in the group exposed to the highest concentration (50 mg F/L) and modulation in the proteomic profile [17,18]. Thus, it is possible to interpret the morphological results presented here in two ways: (1) the molecular changes did not result in changes in the tissues, and/or (2) chronic exposure over 60 days did not allow enough time for any molecular changes to have an effect on the tissues.

Other factors must also be considered, such as the fact that F exposure may occur from multiple sources and can be influenced by the length of exposure and by the specific developmental phases. Thus, other forms of exposure to F should be considered, as well as other time windows, for a closer assessment of the risks of F exposure to these organs. Further research seeking longer exposure times, and at doses representative of human exposure may better reinforce the safety of F use. Finally, it must be emphasized that there is no evidence to support that F can bring any harm to human health when used at recommended levels [35].

## 5. Conclusions

From the results presented, it can be concluded that chronic exposure to F by mice over a 60-day period (from the end of the lactation period to adult life) led to no significant changes in the morphology of adult rat salivary glands. Nonetheless, the present results should be interpreted with caution, considering that chronic F exposure at earlier developmental stages was previously shown to increase the systemic bioavailability of F, as well as to promote proteomic modulations in important cell cycle processes, cytoskeleton regulation, and cellular metabolism. The simultaneous analysis of the present and previous studies cited above clearly point to the need to further address this important research question, whose results might guide future translational research.

## Data Availability

The quantitative and qualitative data used to support the findings of this study are included in the article.

## Supplementary Materials

The following supporting information can be downloaded at: https://www.mdpi.com/article/10.3390/jcm11185373/s1, Table S1: Parametric results of the morphometric analysis of the parenchyma area, stromal area, and total acinar area, of parotid, submandibular and sublingual glands of mice exposed to 10mg F/L and 50mgF/L for 60 days. Results were expressed as mean ± standard error of mean.; Table S2: Nonparametric results of the analysis of the immunostained area fraction of the smooth muscle actin filaments of the myoepithelial cells of the salivary glands of mice exposed to 10mgF/L and 50mg F/L for 60 days. Results are expressed as median and interquartile ranges.
