# Supplementary material for: Fluoride Exposure and Salivary Glands: How Is Glandular Morphology Susceptible to Long-Term Exposure? A Preclinical Study
Source: J Clin Med. 2022 Sep 13;11(18):5373. doi: 10.3390/jcm11185373 (PMC9501535; doi:10.3390/jcm11185373)
Supplement: Supplementary file 1 [file jcm-11-05373-s001.zip › jcm-1853159-supplementary.pdf]

Supplementary Table S1: Parametric results of the morphometric analysis of the parenchyma area, stromal area, and total acinar area, of parotid, submandibular and sublingual glands of mice exposed to 10mgF/L and 50mgF/L for 60 days. Results were expressed as mean ± standard error of mean.

**PAROTID**			
	**Parenchyma area**	**Stromal area**	**Total acinar area**
**Control**	43480 ± 1761μm²	21676 ± 3306μm²	41451 ± 1644μm²
**10mgF/L**	49502 ± 1817μm²	17982 ± 1670μm²	48300 ± 1425μm²
**50mgF/L**	43700 ± 4278μm²	22341 ± 4924μm²	41166 ± 3659μm²

**SUBMANDIBULAR**			
	**Parenchyma area**	**Stromal area**	**Total acinar area**
**Control**	46286 ± 6342μm²	11910 ± 1782μm²	40680 ± 5803μm²
**10mgF/L**	57736 ± 4176μm²	14588 ± 4176μm²	48228 ± 4178μm²
**50mgF/L**	57342 ± 1427μm²	9940 ± 1588μm²	49766 ± 1676μm²

**SUBLINGUAL**			
	**Parenchyma area**	**Stromal area**	**Total acinar area**
**Control**	47535 ± 2210μm²	17348 ± 1488μm²	46868 ± 2256μm²
**10mgF/L**	49336 ± 4177μm²	18362 ± 3805μm²	46191 ± 5074μm²
**50mgF/L**	40123 ± 1436μm²	27201 ± 1436μm²	38952 ± 1461μm²

Supplementary Table S2: Nonparametric results of the analysis of the immunostained area fraction of the smooth muscle actin filaments of the myoepithelial cells of the salivary glands of mice exposed to 10mgF/L and 50mgF/L for 60 days. Results are expressed as median and interquartile deviation.

	**Area Fraction**					
	**Parotid**		**Submandibular**		**Sublingual**	
	**Median**	**Interquartile range**	**Median**	**Interquartile range**	**Median**	**Interquartile range**
**Control**	22.04	16.67	21.34	7.65	5.047	1.887
**10mgF/L**	18.58	5.85	13.79	2.02	3.390	1.703
**50mgF/L**	24.31	10.09	20.92	10	4.651	4.268
